# Enhanced anti-hepatocarcinoma efficacy by GLUT1 targeting and cellular microenvironment-responsive PAMAM–camptothecin conjugate

**DOI:** 10.1080/10717544.2017.1419511

**Published:** 2017-12-28

**Authors:** Pengkai Ma, Yi Sun, Jianhua Chen, Hongpin Li, Hongyu Zhu, Xing Gao, Xinning Bi, Yujie Zhang

**Affiliations:** ^a^ School of Chinese Materia Medica, Beijing University of Chinese Medicine Beijing China; ^b^ Institute of Pharmacology & Toxicology, Academy of Military Medical Sciences Beijing China

**Keywords:** GLUT1 targeting, tumor microenvironment, PAMAM dendrimer, conjugate, anticancer drug

## Abstract

The efficient targeting of drugs to tumor cell and subsequent rapid drug release remain primary challenges in the development of nanomedicines for cancer therapy. Here, we constructed a glucose transporter 1 (GLUT1)-targeting and tumor cell microenvironment-sensitive drug release Glucose–PEG–PAMAM-s-s–Camptothecin-Cy7 (GPCC) conjugate to tackle the dilemma. The conjugate was characterized by a small particle size, spherical shape, and glutathione (GSH)-sensitive drug release. *In vitro* tumor targeting was explored in monolayer (2D) and multilayer tumor spheroid (3D) HepG2 cancer cell models (GLUT1^+^). The cellular uptake of GPCC was higher than that in the control groups and that in normal L02 cells (GLUT1^−^), likely due to the conjugated glucose moiety. Moreover, the GPCC conjugate exhibited stronger cytotoxicity, higher S arrest and enhanced apoptosis and necrosis rate in HepG2 cells than control groups but not L02 cells. However, the cytotoxicity of GPCC was lower than that of free CPT, which could be explained by the slower release of CPT from the GPCC compared with free CPT. Additional *in vivo* tumor targeting experiments demonstrated the superior tumor-targeting ability of the GPCC conjugate, which significantly accumulated in tumor meanwhile minimize in normal tissues compared with control groups. The GPCC conjugate showed better pharmacokinetic properties, enabling a prolonged circulation time and increased camptothecin area under the curve (AUC). These features contributed to better therapeutic efficacy and lower toxicity in H22 hepatocarcinoma tumor-bearing mice. The GLUT1-targeting, GSH-sensitive GPCC conjugate provides an efficient, safe and economic approach for tumor cell targeted drug delivery.

## Introduction

Nanomedicine-based tumor therapeutics has shown potential for overcoming the poor specificity of conventional chemotherapeutics and provides clinicians with treatment alternatives (Hu et al., 2017; Mohamed et al., 2017). The underlying delivery mechanism of nanomedicines, such as Doxil^®^, Abraxane^®^, and Genexol-PM^®^, is based on the enhanced permeability and retention (EPR) effect (Barenholz, 2012; Lammers et al., 2012; Louage et al., 2017). Unfortunately, clinical trials using these nanomedicines have shown limited antitumor effects. The simple passive delivery of nanocarriers to tumor sites is not capable of eliciting significant positive therapeutic responses; the treatment outcomes of nanomedicines are dependent not only on localization but also on sufficient cell drug concentrations (Danhier, 2016; Shi et al., 2016). Thus, effectively deliver chemotherapeutic drugs to tumor cell is emerging as a more important issue.

The most commonly used approach to enhance the delivery efficiency of carriers is the conjugation of targeting ligands that specifically recognize and bind to overexpressed receptors on tumor cell surfaces. As an alternative to receptor-mediated pathways, transporter-mediated pathways have faster transport rates and improved efficiency and specificity (Shao et al., 2014). Facilitative glucose transporter 1 (GLUT1), an important member of the glucose transporter protein (GLUT) family, transports d-glucose across cell membranes (Niu et al., 2014; Wu et al., 2016). Known as Warburg effect, tumor cells consume large quantities of glucose for proliferation (Jiang et al., 2014), which results in the overexpression of GLUT1 by most carcinoma cells. Anticancer drugs designed for targeting GLUT is still in the ascendant in oncology research (Labak et al., 2016). For example, 2-deoxy-2-(18F)-fluoro-d-glucose, as the most convincing example, has been widely adopted for positron emission tomography (PET) imaging for cancer diagnoses (Jadvar, 2016). Glufosfamide, a derivative of ifosfamide mustard conjugated to glucose, has shown promise in treating metastatic pancreatic cancer in advanced clinical trials (Lacombe, 2012; Li et al., 2016). However, highly effective GLUT1-mediated, tumor-targeting nanomedicines have not been widely developed.

Smart controllable drug release from nanocarriers in tumor cells remains another crucial issue. Before nanocarriers are taken up by tumor cells, they release most of the loaded drug into the systemic circulation, ultimately leading to systemic toxicity and poor anticancer efficacy (Karimi et al., 2016; You et al., 2017). Tumor cell microenvironment shows significant differences from it of normal cells (Danhier, 2016; Chen et al., 2017). The intracellular concentration of glutathione (GSH) is approximately 10 mM, which is 5000-fold higher than that in extracellular environments. This significant difference in GSH levels has been explored as a trigger for drug release inside target cells with reductive sensitivity (Stephen et al., 2014; Guo et al., 2015). The introduction of GSH-triggered disulfide cross-links to polymer nanocarriers can stabilize carriers against hydrolytic degradation and efficiently initiate drug release once the carriers are internalized in the target cells (Zou et al., 2016). However, these nanomedicines generally require sophisticated designs and complex fabrication procedures. The few clinically successful nanomedicines have shown that in addition to efficacy and safety, simplicity, and cost play a decisive role in their translation into therapeutic products (Hofmann-Amtenbrink et al., 2015; Hare et al., 2016). Polymeric conjugates may address these issues, since a few chemotherapeutic drugs conjugated with various functional polymers have been approved for clinical trials and even for the market, including N-[2-hydroxylpropyl] methacrylamide (HPMA)-doxorubicin (PK1/FCE28068) (Seymour et al., 2009) and poly-glutamacid (PGA)-SN38 (NK012) (Hamaguchi et al., 2010).

Poly(amido amines) (PAMAMs) are dendrimer polymers characterized by nanosized spherical shapes with 3D tree-like branching structures. Compared with liposomes, micelles, and nanoparticles, PAMAMs have unique advantages, such as monodispersity, controlled synthesis, good biocompatibility, and tunable size. The abundant external terminal groups (i.e. COOH, NH_2_, and OH) enable PAMAMs to be modified with ligands, fluorescent probes, and other functional molecules (Luong et al., 2016). These unique properties provide PAMAM dendrimers advantages in serving as nanocarriers in polymeric conjugate nanomedicines (Labieniec-Watala & Watala, 2014; Sadeghpour, 2015).

**Figure 1.**
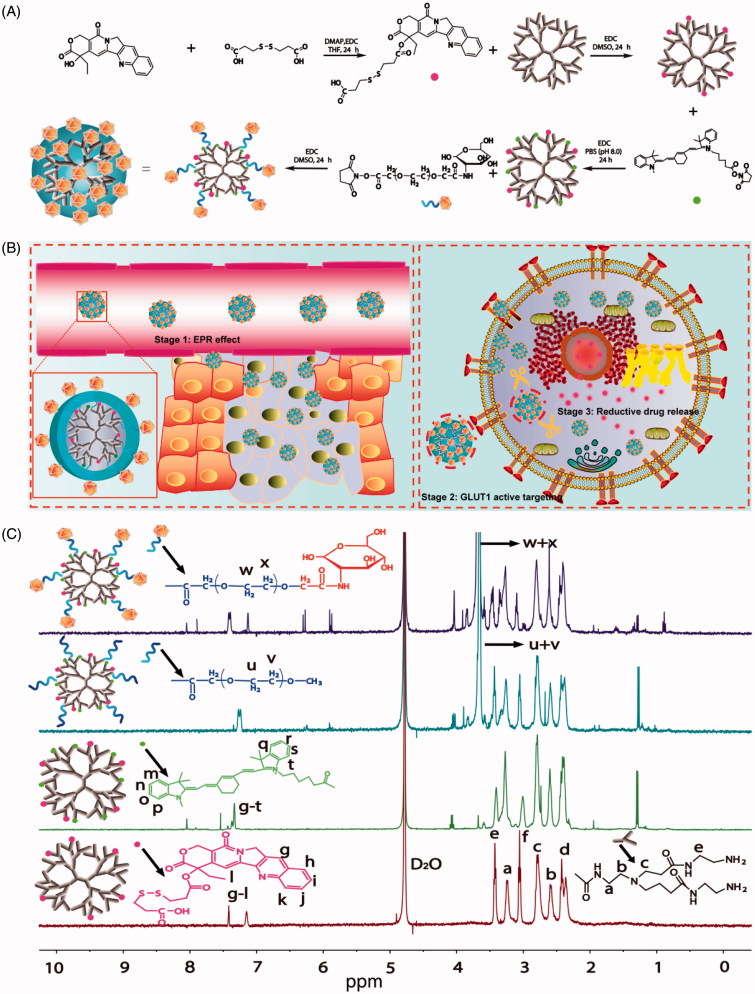
(A) Synthesis schematics for the GPCC conjugate. CPT was endowed with reductive sensitivity of GSH by the introduction of a disulfide bond via an esterification reaction. CPT was then conjugated to the amine-terminated PAMAM dendrimer via an amidation reaction. The Cy7 tracer was used to label PAMAM to observe the distribution of conjugates *in vivo*. Finally, glucose-functionalized PEG was linked to PAMAM, which endowed the conjugate with prolonged circulation and GLUT1-targeting abilities. (B) Schematics of the structure of the GPCC conjugate and the action mechanism of enhanced cancer cell targeting and cellular microenvironment-sensitive drug release. The GPCC conjugate first accumulates in tumor tissue through the EPR effect; then, the GPCC conjugate is recognized and endocytosed by glucose transporter 1 (GLUT1)-overexpressing cells through glucose-GLUT1-specific interactions; the endocytosed conjugate is delivered to the cell cytoplasm, where high GSH concentrations trigger intracellular camptothecin release and finally, free drugs act on the nucleus. (C) ^1^HNMR of different conjugates (from top to bottom: GPCC, MPCC, PCC, and PC).

Herein, we used GLUT1-specific ligands and GSH-sensitive linkers to construct a new PAMAM nanoconjugate to exploit the properties of these various motifs to enhance tumor cell targeting and tumor cell microenvironment-sensitive drug release with the aim of improving anticancer efficacy and reducing the systemic toxicity commonly accompanying chemotherapeutics. A PAMAM dendrimer was employed as the nanocarrier, glucose as targeting ligands, PEG as a linker for prolonging circulation, camptothecin (CPT) as the model drug and Cy7 as an imaging marker to construct a Glucose-PEG-PAMAM-s-s-CPT-Cy7 (GPCC) conjugate (Figure 1(A)). The action mechanism of this nanoconjugate is illustrated in Figure 1(B).

## Materials and methods

### Drugs and reagents

G4 PAMAM dendrimer (10 wt% solution in methanol), glucose, camptothecin, 1-ethyl-3-(3-dimethylaminopropyl) carbodiimide hydrochloride (EDC), 3,3'-dithiodipropionic acid, and 4-dimethylaminopyridine (DMAP) were purchased from Sigma-Aldrich Co., LLC (St. Louis, MO). Glucose–PEG–NHS and mPEG–NHS were obtained from Jiankai Technology Company (Beijing, China). The fluorescent probes Basic Orange 14, Cy7-NHS, and MitoTracker Red were all obtained from FanBo Biochemical Corporation (Beijing, China). Dulbecco’s modified Eagle’s medium (DMEM), 3-(4,5-dimethyl-2-thiazolyl)-2,5-diphenyl-2-*H*-tetrazolium bromide (MTT), and fetal bovine serum (FBS) were purchased from Gibco Life Technology Company (Grand Island, NY). HepG2 and L02 cells were gifts from Dr. Dengke Li of the School of Life Science of Beijing University of Chinese Medicine. Male Kunming mice and Sprague–Dawley rats were obtained from Sibeifu Laboratory Animal Technology Company (Beijing, China).

### Synthesis of camptothecin derivative

To conjugate CPT with PAMAM and enable the conjugates to release drug with the GSH reductive sensitive property, a free carboxyl group and a disulfide bond were introduced to the CPT. 3,3'-Dithiodipropionic acid (492 mg, 2.34 mmol) was dissolved in tetrahydrofuran, DMAP (57.42 mg, 0.47 mmol), and EDC (90.10 mg, 0.47 mmol) were added, and the mixture was stirred for 4 h in an ice bath. Then, camptothecin (CPT, 163.72 mg, 0.47 mmol) was added, and the mixture was stirred for 24 h at room temperature in the dark. The reaction mixture was concentrated by reduced pressure distillation. After the mixture was re-dissolved in dichloromethane, the catalyzers were removed by washing with saturated sodium chloride. The dichloromethane layer was collected and purified by elution with dichloromethane/methanol (100/1) to yield the CPT derivative. The CPT derivative was characterized by ^1^HNMR (Avance 500, Bruker, Switzerland) and high-resolution mass spectroscopy (LTQ-Orbitrap XL, Thermo Fisher Scientific, Waltham, MA) (Figure S1). ^1^HNMR (500 MHz, CDCI_3_/CD_3_OD): *δ* 1.01 (t, 3H, –CH_2_CH_3_), *δ* 2.19–2.25 (m, 2H, –CH_2_CH_3_), *δ* 2.65 (t, 2H, –CH_2_SSCH_2_–), *δ* 2.88 (t, 2H, –CH_2_SSCH_2_–), *δ* 2.94–2.97 (m, 4H, –OCCH_2_CH_2_SSCH_2_CH_2_COOH), *δ* 5.31 (s, 2H, –COOCH_2_–), *δ* 5.40–5.43 (d, 1H, –NCH_2_C–), *δ* 5.62–5.65 (d, 1H, –NCH_2_C–), *δ* 7.58 (s, 1H, –(CH_2_)(CO)NC(C = N)=CH–), *δ* 7.69 (t, 1H, (C_6_H_4_)CH = C(CH_2_)(C = N)–), *δ* 7.85, 8.00, 8.20, 8.55 (t, d, d, s, 1H, 1H, 1H, 1H, phenyl). ESI-QTRAP-MS: *m*/*z* calculated from C_26_H_24_N_2_O_7_S_2_ – H^−^: 539.10249; observed: 539.09412.

### Synthesis of PAMAM–CPT conjugate

CPT derivative (188.69 mg, 0.35 mmol) dissolved in DMSO was added to DMAP (43 mg, 0.35 mmol), EDC (67 mg, 0.35 mmol), and NHS (67 mg, 0.35 mmol) to synthesize the CPT active ester. The mixture was reacted for 24 h at room temperature and used directly without further purification. PAMAM (248.76 mg, 1.75 × 10^−2 ^mmol) was added, and the mixture was reacted for another 24 h. The free CPT derivative was removed by dialysis against water for 24 h; the water was refreshed every 8 h. The final product, PAMAM–CPT (PC), was concentrated with ultrafiltration tube, lyophilized and characterized by ^1^HNMR and UV–Vis spectroscopy.

### Synthesis of PAMAM–CPT–Cy7 conjugate

PC (100 mg, 5.94 × 10^−3 ^mmol) and Cy7-NHS (21.29 mg, 2.97 × 10^−2 ^mmol) were dissolved in PBS (pH 8.0) and vigorously agitated for 24 h in dark (Ma et al., 2017). Free Cy7-NHS was removed by dialysis against water for 24 h, and the residue was concentrated, lyophilized and characterized by ^1^HNMR and UV–Vis spectroscopy.

### Synthesis of mPEG/glucose–PEG–PAMAM–CPT–Cy7 conjugate

PAMAM–CPT–Cy7 (PCC, 100 mg, 5.20 × 10^−3 ^mmol) was dissolved in PBS (pH 8.0); glucose-PEG-NHS (1300.03 mg, 0.26 mmol) was added, and the mixture was reacted for 24 h at room temperature. The reaction mixture was purified to remove free PEG by dialysis against water for 24 h, and the final product, Glucose–PEG–PAMAM–CPT–Cy7 (GPCC), was concentrated, lyophilized, and characterized by ^1^HNMR and UV–Vis spectroscopy. The mPEG-NHS was also reacted with PCC to yield an mPEG–PAMAM–CPT–Cy7 (MPCC) conjugate as control (He et al., 2011).

### Characterization of conjugates

#### Size, zeta potential, and morphology

The particle sizes and zeta potential of different conjugates at 10 mg/ml were determined using a dynamic light scattering (DLS) particle size analyzer (Nicomp 380 Zeta Potential/Particle Sizer, Santa Barbara, CA). Following staining with 2% sodium phosphotungstate solution, the conjugate morphologies were observed using transmission electron microscopy (TEM) at an accelerating voltage of 100 kV (JEM 1400 JOEL, Akishima City, Tokyo Prefecture, Japan).

#### In vitro drug release

A dialysis method was used to determine the *in vitro* CPT release of conjugates. Conjugates at concentrations of 1 mg/ml were placed in dialysis bags (MWCO 3500 Da) and immersed in 50 ml PBS (pH 7.4) containing different concentrations of GSH (0 μM, 10 μM and 10 mM) to mimic various cellular microenvironment conditions. The drug release was conducted at 100 rpm and 37 °C. During dialysis, 1 ml aliquots were withdrawn from the release medium at predefined intervals, and 1 ml of fresh release medium was added to maintain a constant total volume. The CPT concentrations were determined by HPLC.

### *In vitro* cell targeting evaluation

HepG2 and L02 cells were seeded into 96-well culture plates at a density of 1.2 × 10^4^ cells/well and incubated for 24 h. After the confluency and morphology were checked, 1 μM conjugates (calculated from CPT) were added to each well and co-incubated with the cells for different times (1, 4, and 8 h). To confirm the specificity of the GPCC conjugate, another group of HepG2 cells was preincubated with 2.5 mM d-glucose (d-GLU) for 4 h to block the GLUT1 transporter. The D-GLU was a substrate as well as inhibitor of GLUT1. Then, the GPCC conjugate was co-incubated with the cells for 4 h. At the end of the incubation time, the conjugate solutions were withdrawn from the wells, and the cells were washed three times with cold PBS. After fixation with 4% paraformaldehyde, the cells were qualitatively analyzed by fluorescence microscopy (IX71, Olympus, Tokyo, Japan). After trypsinization and re-suspension of the cells in PBS (pH 7.4), the fluorescence intensity was quantitatively analyzed by flow cytometry (BD FACSAria III, Piscataway, NJ).

Besides that, a multicellular tumor spheroids (MCTS) model, which was more suitable for mimicking the tumor microenvionment, was also employed as a supplement to monolayer cell model for better estimating *in vitro* targeting efficacy (Kunz-Schughart, 1999). MCTS model was established by the liquid overlay method as described before (Friedrich et al., 2008). Briefly, HepG2 cells were seeded in 96-well plates coated with 2% agarose at 2 × 10^3^ cells per well and incubated for 5 d. Then, MCTS were treated with 200 µL of 1 μM PCC, MPCC, GPCC for 8 h with free CPT as control. Thereafter, MCTS were collected by centrifugation, washed with PBS, and observed by confocal laser scanning microscope (CLSM) (Olympus FV1000, Tokyo, Japan) at 100 µm depth of the MCTS (about the middle section of the sphere).

### Subcellular localization

HepG2 and L02 cells were seeded on cover glasses embedded in 24-well culture plates at densities of 5 × 10^4^ cells/well. After 24 h, conjugates were added at a 1 μM CPT concentration and co-incubated with the cells for 4 h at 37 °C. Then, after the conjugate solutions were removed, the cells were treated with 50 nM MitoTracker deep red for 30 min and 10 mM Basic Orange 14 for 10 min to stain mitochondria and nuclei, respectively (Paleos et al., 2016). Then, the cells were fixed with 4% paraformaldehyde, washed three times with ice-cold PBS (pH 7.4), and visualized using CLSM.

### Cytotoxicity, cell-cycle analysis, and apoptosis assay

The cytotoxicity of different conjugates on HepG2 and L02 cells was determined using 3-(4,5-dimethyl-2-thiazolyl)-2,5-diphenyl-2-*H*-tetrazolium bromide (MTT) method. The cells were seeded in 96-well culture plates at a density of 5 × 10^3^ cells/well. Then the cells were treated with conjugates and free CPT at CPT concentrations ranging from 3 nM to 20 μM at 37 °C for 24 h when 60–70% confluence was reached. After that, the drug solution was replaced with MTT at a concentration of 5 mg/mL, and co-incubated with cells for another 4 h. The supernatant was discarded and adding with 150 μL DMSO to dissolve the MTT-formazan. The absorbance at 492 nm was recorded using a microplate reader.

The cell-cycle percentage of HepG2 was analyzed using flow cytometer. The cells were seeded in 6-well plates at a concentration of 1 × 10^6^ cells/well and treated with 1 ml conjugates solution at 1 μM CPT when cells growing to the logarithm stage. After 24 h, the cells were trypsinized, washed with PBS, and fixed with 4 °C pre-cooling 70% ethanol overnight at −20 °C. Then the cells were washed two times with PBS and treated with 0.5 mg/mL RNAse at 37 °C for 30 min. Finally, the cells were stained with propidium iodide (PI) solution for 20 min and determined by flow cytometer.

The apoptosis induction effect of conjugates on HepG2 cells was detected by Annexin V-FITC/PI double staining method. Briefly, the cells were seeded in 6-well plates at a concentration of 1 × 10^6^ cells/well and treated with 1 ml conjugates solution at 1 μM CPT when cells growing to the logarithm stage. After 24 h, the cells were trypsinized (without EDTA), washed with PBS (containing 2% BSA), stained by 5 μL of Annexin V-FITC and 5 μL of 100 μg/mL PI, and re-suspended in 500 μL of binding buffer. After 15 min incubation, cells were determined by flow cytometer.

### *In vivo* targeting evaluation

#### Tumor implantation

An H22 tumor-bearing mice model was established based on previously reported method (Li et al., 2014). Ascitic fluid containing H22 cells was extracted and diluted with PBS (pH 7.0) to 1 × 10^7^ cells/ml. The mice (20–22 g) were anesthetized with 5% chloral hydrate and subcutaneously injected with 0.2 ml of cell suspension for armpit tumor implantation. All animal studies were approved by the China Animal Care and Use Committee and carried out according to the Guide for the Care and Use of Laboratory Animals of Beijing University of Chinese Medicine.

#### In vivo imaging

Eighteen tumor-bearing mice were randomly divided into three groups, i.e. the PCC, MPCC, and GPCC conjugate groups. Mice were intravenously injected with conjugates at 1 mg/kg CPT concentration. Following anesthetization with 5% chloral hydrate, the mice were observed at 1, 2, 4, 8, and 12 h post-injection using an *in vivo* imaging system (Carestream, Rochester, NY). Afterward, the tumor-bearing mice were immediately sacrificed by carbon dioxide asphyxiation. Subsequently, major organs (such as tumor, liver, spleen, brain, heart, kidney, and lung) were harvested and visualized.

### Pharmacokinetic study

Twenty-four rats (200 ± 20 g) were randomly divided into four groups and were intravenously administered 1.5 mg/kg doses of CPT, PCC, MPCC, or GPCC. The rats were fasted for 12 h with free access to water before the experiment. Briefly, 0.5 ml of blood was collected in centrifuge tubes at predefined times of 0.083, 0.33, 0.5, 1, 2, 4, 8, 12, and 24 h. Blood samples were centrifuged at 15,000 rpm for 15 min at 4 °C to isolate plasma fractions. Then, 100 μL of methanol was added to the plasma, and the mixture was extracted with 2 mL hexane and 1 mL dichloromethane. The mixture was vortexed for 10 min and centrifuged at 15,000 rpm for 15 min, and the supernatant was collected and dried using a stream of nitrogen gas. The resultant residue was redissolved in 200 μL methanol and stored at −20 °C until further analysis.

### Anti-tumor efficacy

The tumor-bearing mice were randomly divided into five groups (10 per group). The control group mice were administered saline. The other four groups were administered free CPT, PCC, MPCC, and GPCC via tail vein injections at a CPT dose of 1 mg/kg on days 0, 2, 4, 6, 8, and 10. Body weights were recorded daily. At day 12, four mice of each group were sacrificed by carbon dioxide asphyxiation. Tumors, livers and spleens were collected and fixed with 10% paraformaldehyde for 48 h. These tissues were cut into 5 mm sections and processed for H&E staining. The remaining rats in each group were also sacrificed, and the tumors were collected and weighed.

### Statistical analysis

Data are presented as the mean ± standard deviation of at least six repeated samples. Differences between groups were analyzed using SPSS statistical software (SPSS, Chicago, IL). Differences were considered significant at *p* < .05.

## Results and discussion

*Synthesis of* Conjugates: The successful synthesis of the conjugates was confirmed by ^1^HNMR and UV–Vis spectroscopy. As shown in Figure 1(C), the peaks at *δ* (ppm) = 2.2–3.6 of all conjugates corresponded to the methylene protons of the branching units of PAMAM. The peak at *δ* (ppm) = 3.6 ppm in the spectra of the MPCC and GPCC conjugates belonged to the methylene protons of PEG. The PEG number was calculated to be twenty by comparing the number of protons of PAMAM to the methyl groups of PEG, as previously described (Tang et al., 2012). The peaks at *δ* (ppm) = 7–8 of the PC conjugate could be assigned to the aromatic groups (6 protons) of CPT. Similarly, the peaks at *δ* (ppm) = 7–8 of the other conjugates were assigned to combinations of the aromatic groups of CPT and Cy7 (Khandare et al., 2006). The UV scanning spectra of the conjugates were observed at 380 nm and 720 nm, respectively, which were consistent with the maximum absorption wavelengths of CPT and Cy7, respectively (Figure S2). The average numbers of CPT and Cy7 molecules conjugated to PAMAM were 5 and 4, respectively, as determined by calibration to free CPT and Cy7.

### Characterization of conjugates

**Figure 2.**
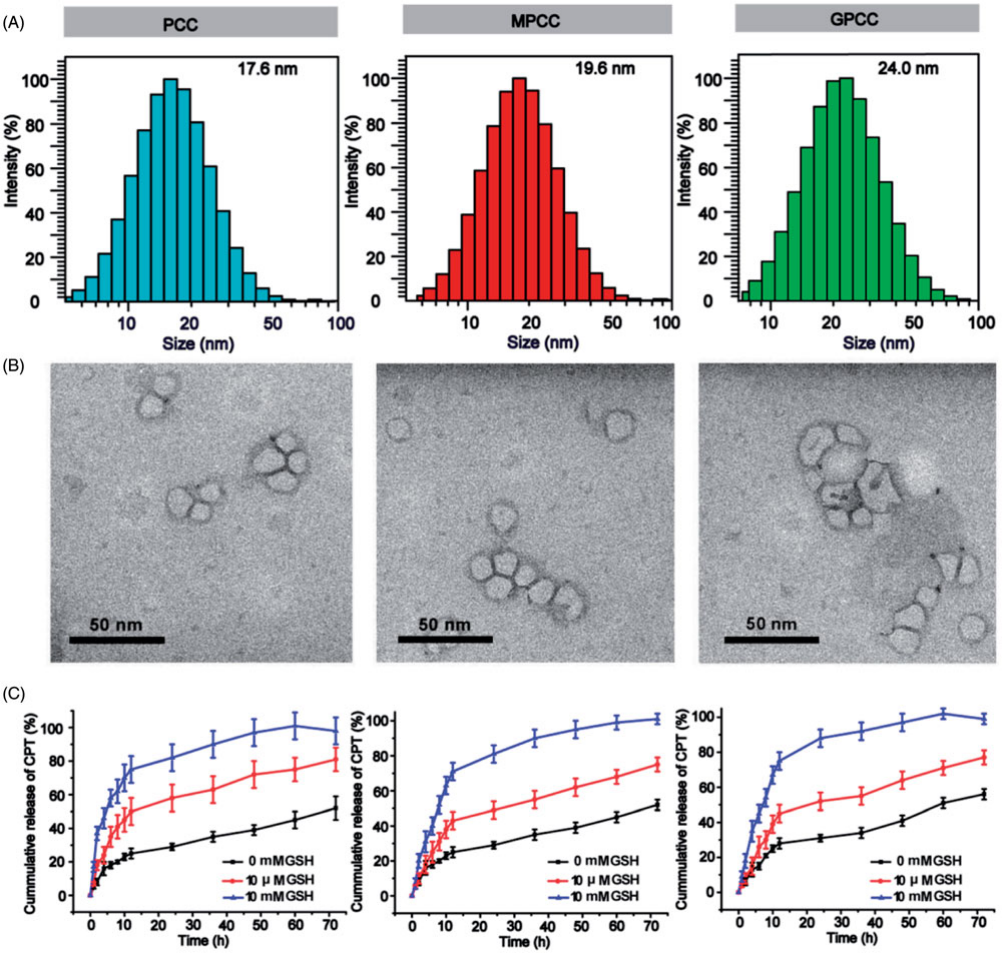
Characteristics of different conjugates. (A) Size distribution of conjugates. (B) Transmission electron microscopy images of conjugates. (C) *In vitro* CPT release from conjugates under different reductive environments.

**Figure 3.**
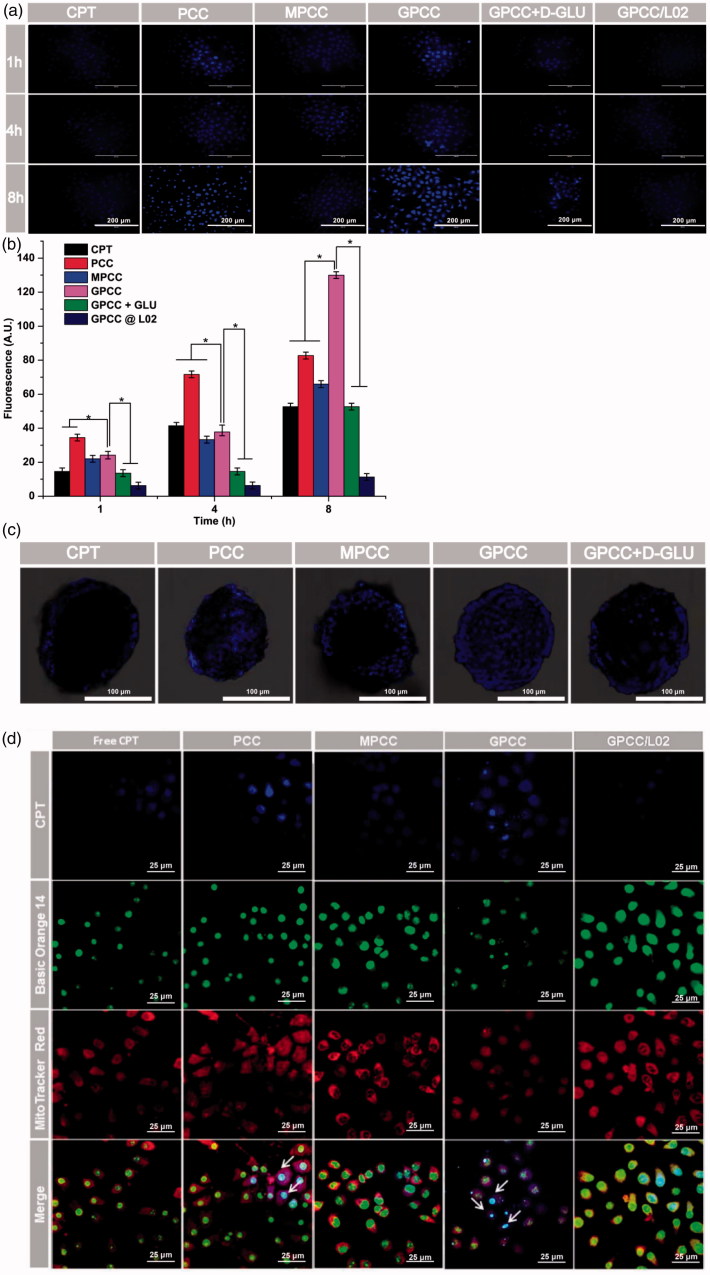
*In vitro* targeting evaluation of the GPCC conjugate for HepG2 and L02 cells. (A) Qualitative cellular uptake of conjugates by GLUT1^+^ HepG2 cells compared with GLUT1^-^ L02 cells at different times. (B) Quantitative cellular uptake of conjugates by HepG2 and L02 cells at different times. (C) Images of HepG2 MCTS treated with conjugates at 1 μM for 8 h, images were captured at a *z*-depth of 100 µm of each MCTS. The GPCC conjugate exhibited significantly enhanced targeting of GLUT1^+^ HepG2 cells compared with that of other conjugates and that for GLUT1^−^ L02 cells. (D) Subcellular localization of conjugates in GLUT1^+^ HepG2 cells and GLUT1^-^ L02 cells visualized by confocal fluorescence microscopy. Cells co-incubated with different conjugates at a CPT concentration of 1 μM for 4 h. The white arrows indicate co-localization of the conjugates and the nuclei. *p < .05.

The particle sizes of PCC, MPCC, and GPCC were 17.6 nm, 19.6 nm, and 24.0 nm, respectively, which indicated that the particle size increased as more molecules were conjugated to PAMAM (Figure 2(A)). The morphology of the conjugates as observed by TEM showed approximate spherical shapes (Figure 2(B)). The particle sizes as determined by TEM were 11.5 nm, 12.2 nm, and 13.1 nm, which were slightly smaller than those observed by DLS. These differences may be due to the hydrated corona of conjugates in water; and the larger particles had a greater contribution to the DLS results (Jin et al., 2014). The CPT release profiles are shown in Figure 3(C). Different release media were used to mimic the various physiological environments. Approximately 75% of the conjugated CPT was released in 12 h with less than 10 mM GSH, and less than 50% and 25% were released with less than 10 μM GSH and 0 mM GSH, respectively. Moreover, PCC showed a slightly increased release rate compared with those of MPCC and GPCC, which may have been due to the PEG layers of MPCC and GPCC hindering interactions between GSH and disulfide bonds. The MPCC and GPCC conjugates showed similar drug release profile due to the similar structure of them. These results demonstrated that the conjugates were sensitive to GSH reduction. The zeta potential values of the conjugates were positive and decreased with other molecules conjugation (Figure S3).

### *In vitro* cell targeting

#### Cellular uptake

HepG2 cells (GLUT1-overexpressing) and L02 cells (GLUT1-lowexpressing) were used as cell models to evaluate the GLUT1-targeting ability of the conjugates. As shown in Figure 3(A), the qualitative cellular uptake of different conjugates observed by fluorescence microscopy was time dependent in the HepG2 and L02 cells. The cellular uptake of GPCC was greater than those of MPCC and PCC in the HepG2 cells at the same time points. However, the cellular uptake of PCC was greater than that of MPCC, which may have been caused by the more positive charge of PCC. The quantitative analyses showed that the uptake of GPCC was approximately two-fold higher than that of MPCC in the HepG2 cells, which was consistent with the images captured by fluorescence microscopy (Figure 3(B)). Additionally, cellular uptake could be blocked by d-glucose, which is a specific substrate of GLUT1 (Singh, 2017). The cellular uptake of GPCC in HepG2 cells was more than that in L02 cells. These results showed that GPCC had better targeting capabilities for GLUT1-overexpressing cells due to transporter-mediated endocytosis.

To further evaluate the targeting ability of conjugates in MCTS, fluorescence distributed in the middle section of the spheroids (100 µm depth) were obtained by CLSM. As shown in Figure 3(C), both PCC and GPCC conjugations exhibited obviously stronger cellular uptake than the MPCC conjugate. This demonstrated that the PCC conjugate possessed stronger penetration ability due to the positive charge of PAMAM dendrimer. Additionally, the GPCC conjugate had better targeting ability due to the interaction between glucose and GLUT1, and the specific interaction could be blocked by glucose.

#### Subcellular localization of conjugates

CPT, as a selective inhibitor of topoisomerase I, is involved in relieving supercoiling structure that emerges during DNA replication and transcription (Das et al., 2016). To evaluate whether the GPCC conjugate could deliver CPT into cells and the distribution of it in cells, a CLSM was employed to visualize the intracellular trafficking of the conjugates. As shown in Figure 3(D), free CPT was hardly observed in the cells at 4 h; however, the PCC conjugate accumulated in the nuclei of the HepG2 cells due to the non-specific cellular uptake of the highly positively charged PAMAM. This result was consistent with other studies that considered PAMAM to be a superior cell transfection agent. Moreover, for the MPCC conjugate, the PEG outer layer slowed the cellular uptake due to the hydrophilic property of PEG and it covered the positive charges of PAMAM. The GPCC conjugate accumulated in HepG2 nuclei more than the MPCC conjugate, which may be due to specific interactions between the GPCC glucose and GLUT1. These results indicated that the GPCC conjugate could efficiently deliver CPT into tumor cells but not normal cells.

### Cytotoxicity, cell cycle, and apoptosis assay

**Figure 4.**
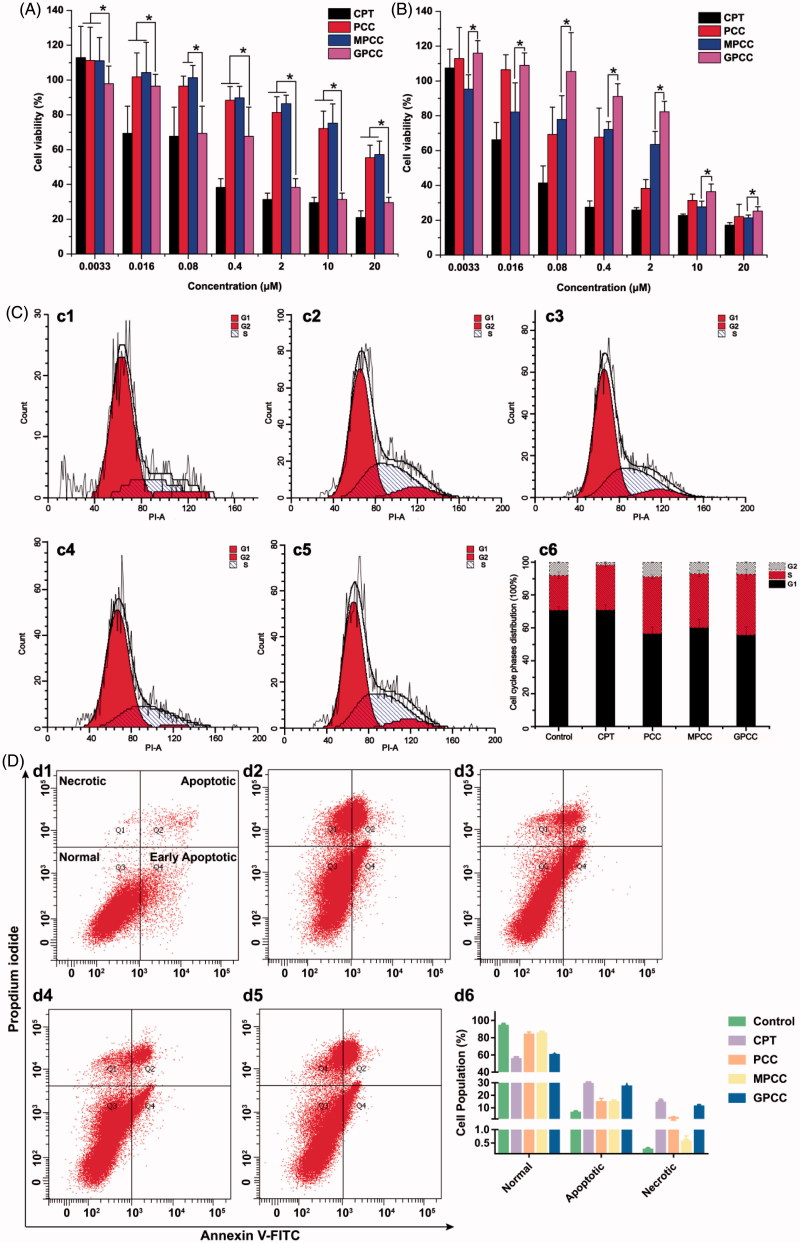
Cytotoxicity of conjugates to GLUT1^+^ HepG2 cells (A) compared with GLUT1^-^ L02 cells (B) incubated for 24 h; (C) effects of treatment with free CPT, PCC, MPCC, and GPCC on the cell-cycle progression of HepG2 cells; (c1) control cells; (c2) cells treated with free CPT, (c3) PCC, (c4) MPCC, and (c5) GPCC at CPT concentration of 1 μM. (c6) The percent of cell-cycle distribution after treatment with conjugates for 24 h. (D) Apoptosis induction effect of various conjugates on HepG2 cells determined by Annexin V-FITC/PI double staining method; (d1) control cells; (d2) cells treated with free CPT, (d3) PCC, (d4) MPCC, and (d5) GPCC at CPT concentration of 1 μM. *p < .05.

The anti-proliferative effects of different conjugates were evaluated using HepG2 and L02 cells. As shown in Figure 4(A,B), the GPCC conjugate exhibited the strongest inhibitory effect on the proliferation of HepG2 cells; this finding may be attributable to the higher uptake rates of GLUT1-overexpressing HepG2 cells. However, the cytotoxicity of GPCC was lower than that of free CPT, which could be explained by the lower cellular drug concentration than free CPT solution. This phenomenon was also observed in the L02 cells. Moreover, the GPCC and MPCC conjugates showed comparable cytotoxicity, which were lower than that of PCC in L02 cells. Thus, the GPCC conjugate had a higher cytotoxicity for GLUT1-overexpressing cancer cells and a lower cytotoxicity for normal cells and showed a superior targeting ability compared with that of the PCC and MPCC conjugates.

CPT, a highly selective topoisomerase I inhibitor, could inhibit cell proliferation through cell cycle arrest. To investigate the action mechanism of cytotoxic effects, various CPT conjugates effects on the cell-cycle progression of HepG2 cells was assessed by flow cytometer. As shown in Figure 4(C), the cell-cycle percentage distribution showed that 21.30% of the control cells were in the S phase, while the S phase percentage increased to 27.49%, 34.7%, 32.94%, and 37.15% for free CPT, PCC, MPCC, and GPCC, respectively. These results revealed that all groups induced the arrest of S phase cell cycle at different levels, and among them the GPCC conjugate exhibited a superior efficacious S phase arrest. It could be inferred that that the glucose increased endocytosis, and GSH responsive drug release enhanced S phase cell-cycle arrest, which resulted in higher in vitro cytotoxicity of CPT. However, the level of S phase did not correlate with the cell viability among different groups. So the apoptosis induction effect of various conjugates was further evaluated on HepG2 cells. As shown in Figure 4(D), the apoptotic cells (early apoptotic cells plus late apoptotic cells) increased obviously in the GPCC group, compared with PCC and mPCC groups (*p* < .05). The free CPT showed the highest apoptosis induction effect, which was similar with the cytotoxicity result. Particularly, all groups showed increased necrotic cells compared with the control group (*p* < .05). And the necrosis induction effect was also correlating with the cell viability of different groups. These results indicated that the cytotoxic mechanism of the conjugates might be a comprehensive effect of cell-cycle arrest, cell apoptosis induction, and cell necrosis induction.

### *In vivo* targeting evaluation

**Figure 5.**
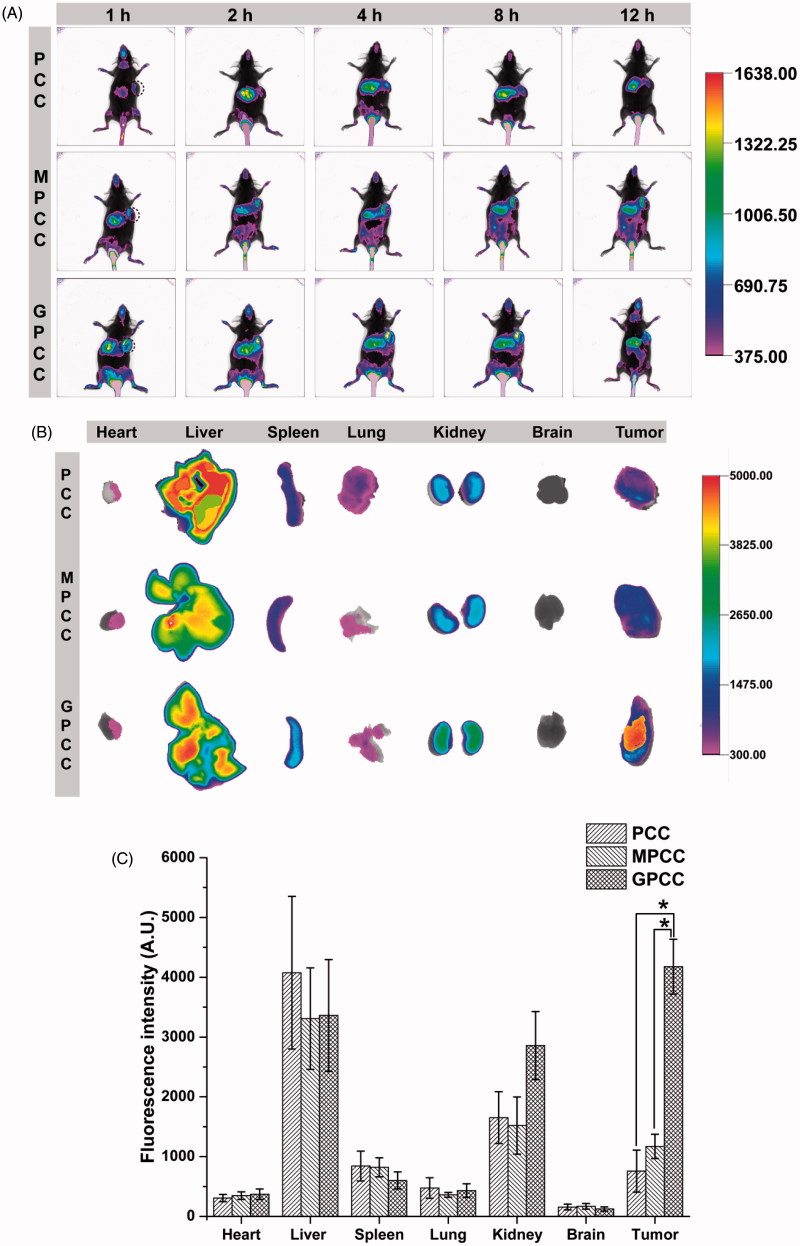
*In vivo* tumor targeting evaluation of different conjugates visualized by *in vivo* imaging system. (A) Real-time *in vivo* imaging distribution of conjugates in H22 tumor-bearing mice at 1, 2, 4, 8, and 12 h after the intravenous injection of conjugates; (B) *ex vivo* imaging distribution of conjugates in tumors and major organs harvested from the tumor-bearing mice at 12 h; and (C) fluorescence intensity of the excised organs at 12 h. *p < .05.

Cy7, a near-infrared fluorescence probe, was conjugated to PAMAM to track the distribution of conjugates in tumor-bearing mice. As shown in Figure 5(A), the GPCC and MPCC conjugates accumulated at tumor sites 1 h post injection, reaching the maximum distribution at 4 h. In contrast, a slight fluorescence could be observed in tumor sites after treatment with the PCC conjugate. *Ex vivo* imaging was also used to confirm the distribution of the conjugates in tissues. The GPCC conjugate exhibited the highest fluorescence intensity in tumor sites, which was approximately 10-fold higher than that of the PCC conjugate and five-fold higher than that of the MPCC conjugate (Figures 5(B,C)). Moreover, other organs (except the liver) showed lower fluorescence intensities, which demonstrated the superior *in vivo* tumor targeting of the GPCC conjugate. For the PCC conjugate, fluorescence was mainly observed at the liver, spleen, and kidney instead of the tumor site.

### Pharmacokinetic study and anti-tumor efficacy

#### Pharmacokinetic study

**Figure 6.**
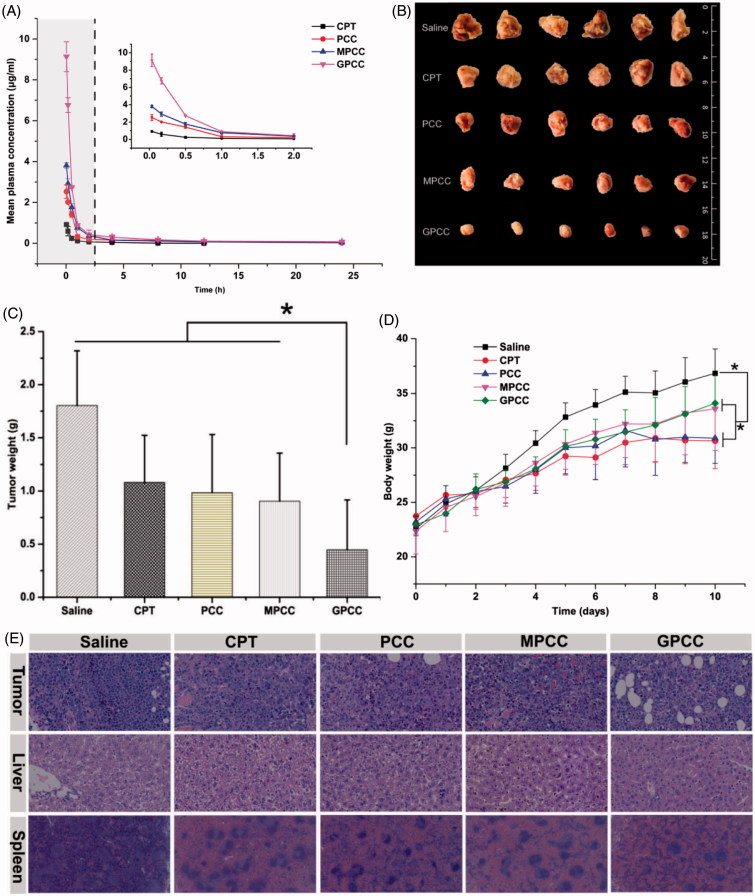
(A) Mean plasma drug concentration–time profiles of CPT in rats after intravenous injection of conjugates at the CPT dose of 1.5 mg/kg; *in vivo* antitumor efficacy of different conjugates in H22 tumor-bearing mice after intravenous administration of CPT at 1 mg/kg. (B) Body weight change during treatment, (C) image, and (D) weight of tumors obtained from mice at the end of experiment, and (E) H&E staining of tumor, liver, and spleen tissues. The data are means ± SD (*n* = 6), Data are shown as mean ± SD (*n* = 6). *p < .05.

Due to the extremely low solubility of CPT, the clinical injection dosages of CPT are limited. Therefore, a CPT suspension was used as a control to compare the pharmacokinetics of the CPT conjugates (Huarte et al., 2016). As shown in Figure 6(A), the clearance of CPT from blood for the CPT conjugates was significantly delayed. The maximum plasma drug concentrations of GPCC, MPCC, and PCC conjugates were 9.13 μg/mL, 3.81 μg/mL, and 2.53 μg/mL, respectively, whereas only 0.92 μg/mL was detected for the CPT suspension at 5 min post-intravenous administration. The pharmacokinetic parameters were calculated using DAS 2.0 software (SPPS Inc., Chicago, IL) with a two-compartment model and are summarized in Table 1. The area under the curve of GPCC was 7.87 mg/L*h, which was 1.68-fold, 2.19-fold, and 10.52-fold higher than that of the MPCC conjugate, the PCC conjugate, and the CPT suspension, respectively. The t_1/2_ of GPCC increased by approximately 1.7-fold, and the mean residence time (MRT) increased approximately two-fold compared with that of the CPT suspension, which indicated that the retention time was prolonged and systemic extravasation was limited. Compared with the CPT suspension, the CPT conjugates had a prolonged retention time and larger AUC. Thereafter, this benefited tumor targeting effects, which was in accordance with the *in vivo* targeting evaluation study.

**Table 1.** Pharmacokinetic parameters of conjugates and CPT suspension after intravenous administration of the 1.5 mg CPT/kg dose.

Parameter	Units	CPT	PCC	MPCC	GPCC
AUC(0–*t*)	mg/L × h	0.748 ± 0.092	3.594 ± 0.665	4.677 ± 0.654	7.865 ± 1.037
AUC(0–∞)	mg/L × h	0.753 ± 0.092	4.038 ± 0.505	4.895 ± 0.62	8.25 ± 0.662
MRT(0–*t*)	h	2.402 ± 0.288	5.042 ± 0.225	4.341 ± 0.9	4.206 ± 0.442
MRT(0–∞)	h	2.591 ± 0.291	9.566 ± 6.991	5.81 ± 1.141	6.016 ± 2.83
*T*_max_	h	0.033	0.033	0.033	0.033
CL	L/h/kg	2.014 ± 0.225	0.376 ± 0.046	0.31 ± 0.037	0.183 ± 0.014
V	L/kg	10.564 ± 1.176	5.104 ± 3.531	3.207 ± 1.638	1.881 ± 1.382
*C*_max_	mg/L	0.917 ± 0.05	2.53 ± 0.33	3.807 ± 0.144	9.132 ± 0.734
*t*_1/2_	h	0.165 ± 0.134	0.398 ± 0.045	0.258 ± 0.027	0.28 ± 0.027

AUC: area under the curve; MRT: mean retention time; *t*_1/2_: half-life; CL: clearance; V: apparent volume of distribution; *C*_max_: maximum drug concentration.

* *p* < .05 compared with the CPT group,

# *p* < .05 compared with the MPCC group.

#### Anti-tumor efficacy

The therapeutic effects of the GPCC conjugate in treating liver tumors were explored in H22 tumor-bearing Kunming mice. Kunming mice have more normal physiological states than nude mice and were thus more suitable for this particular tumor strain (Yang et al., 2017). As shown in Figures 6(B,C), compared with the normal saline group, other groups with drug administration showed tumor inhibition effects. It could be explained that the conjugates exhibited relatively longer retention time and slower drug release profile, which enhanced the delivery of more CPT drugs to the tumor site, resulting in improved anti-tumor efficacy. The GPCC conjugate group showed significantly better tumor inhibition rates than the CPT suspension group and the other conjugate groups throughout the treatment (*p* < .05). The tumor weight of the GPCC conjugate was significantly less than that of the MPCC conjugate (*p* < .05). This result may have been due to the better active targeting ability stemming from the specific interactions between glucose and GLUT1.

The potential toxicities of conjugates were evaluated by the body weight change of mice. As shown in Figure 6(D), the body weight increases for all drug-treated groups were less than that of the normal saline group; the CPT group showed the smallest increase. The mice treated with the GPCC conjugate exhibited similar body weight increases as those of the saline group, which demonstrated the minimal toxicity of all conjugate groups. Histological analyses using H&E staining, as shown in Figure 6(E), revealed that the GPCC conjugate caused widespread necrosis in tumor tissues and limited damage to the liver and spleen. In contrast, the CPT injection and PCC conjugate caused significant hepatotoxicity and spleen toxicity.

## Conclusions

Three novel CPT conjugates, PCC, MPCC, and GPCC were successfully synthesized, and the GPCC conjugate was designed to deliver CPT into GLUT1-overexpressing tumor cells. These conjugates significantly improved the CPT solubility, and possessed small particle sizes, and GSH-reductive drug release properties. *In vitro* cell imaging demonstrated that the GPCC conjugate possessed better ability to target GLUT1-overexpressing HepG2 cells and had a lower cellular uptake by normal L02 cells. *In vivo* imaging and pharmacokinetic studies revealed that three conjugates had varying degrees of prolonged blood circulation time and tumor-targeting abilities. The GPCC conjugate exhibited the best antitumor effect in H22 liver cancer mice models and exhibited the lowest toxicity. Therefore, the GLUT1-targeting, GSH reductive sensitive PAMAM–CPT conjugate can be served as an attractive platform for on demand, anticancer drug delivery, and it is a promising novel nanomedicine for liver cancer treatment.

## Supplementary Material

IDRD_Zhang_et_al_Supplemental_Content.docx
