# Supplementary material for: Enhanced anti-hepatocarcinoma efficacy by GLUT1 targeting and cellular microenvironment-responsive PAMAM–camptothecin conjugate
Source: Drug Deliv. 2017 Dec 28;25(1):153–65. doi: 10.1080/10717544.2017.1419511 (PMC6058575; doi:10.1080/10717544.2017.1419511)
Supplement: IDRD_Zhang_et_al_Supplemental_Content.docx [file IDRD_A_1419511_SM1461.docx]

Professor Stephen von Tetzchner PhD (Corresponding Author)

Email: s.v.tetzchner@psykologi.uio.no

Affiliation 1:

University of Oslo, Psychology, P.O. Box 1094 Blindern, Oslo, NO-0317 Norway

-------------------------------------------------

Dr Kaisa Launonen

Email: kaisa.launonen@helsinki.fi

Affiliation 1:

University of Helsinki, 3Institute of Behavioural Sciences, Siltavuorenpenger 5 A, P.O.Box 9, University of Helsinki, 00014 Finland

-------------------------------------------------

Dr Beata Batorowicz Dr

Email: bbatorow@gmail.com

Affiliation 1:

Queen's University, Faculty of Heath Sciences, School of Rehabilitation Therapy, 31 George Street, Kingston, Ontario, K7L 3NG Canada

-------------------------------------------------

Dr Leila Regina d’Oliveira de Paula Nunes

Email: leilareginanunes@terra.com.br

Affiliation 1:

Universidade do Estado do Rio de Janeiro, Rio de Janeiro, 20550-900 Brazil

-------------------------------------------------

Dr Cátia Crivelenti de Figueiredo Walter

Email: catiawalter@gmail.com

Affiliation 1:

Universidade do Estado do Rio de Janeiro, Rio de Janeiro, 20550-900 Brazil

-------------------------------------------------

Dr Judith Oxley

Email: oxleyjd55@gmail.com

Affiliation 1:

University of Louisiana at Lafayette, Communicative Disorders, P.O. Box 43170, Lafayette, 70503-2014 United States

-------------------------------------------------

Dr Munique Massaro

Email: munique_massaro@yahoo.com.br

Affiliation 1:

Rio Claro Municipality, Rio Claro, 08710240 Brazil

-------------------------------------------------

Ms Kristine Stadskleiv

Email: kstadskl@ous-hf.no

Affiliation 1:

Oslo Universitetssykehus, Department of Child Neurology, P.O. 4956, Oslo, 0424 Norway

-------------------------------------------------

Dr Chih-Kang Yang

Email: ckyangs@gmail.com

Affiliation 1:

National Dong Hwa University Hua-Shih College of Education, Hualian, 97003 Taiwan

-------------------------------------------------

Dr Débora Deliberato

Email: delibera@marilia.unesp.br

Affiliation 1:

UNESP, Special Education, Rua Guanás, 70 ap154, Marilia, 17502560 Brazil
